# Destruction of Carbon and Glass Fibers during Chip Machining of Composite Systems

**DOI:** 10.3390/polym15132888

**Published:** 2023-06-29

**Authors:** Dora Kroisová, Štěpánka Dvořáčková, Artur Knap, Tomáš Knápek

**Affiliations:** Department of Material Science, Department of Machining and Assembly, Faculty of Mechanical Engineering, Technical University of Liberec, 461 17 Liberec, Czech Republic

**Keywords:** carbon fibers, glass fibers, composite systems, machining, destruction

## Abstract

Composite materials with carbon and glass fibers in an epoxy matrix are widely used systems due to their excellent mechanical parameters, and machining is a standard finishing operation in their manufacture. Previous studies focused exclusively on the characteristics of the fibers released into the air. This work aimed to analyze the nature of the material waste that remains on the work surface after machining. The dust on the work surface is made up of fibers and a polymer matrix, and due to its dimensions and chemical stability, it is a potentially dangerous inhalable material currently treated as regular waste. The smallest sizes of destroyed carbon fibers were generated during drilling and grinding (0.1 μm), and the smallest glass fiber particles were generated during milling (0.05 μm). Due to their nature, carbon fibers break by a tough fracture, and glass fibers by a brittle fracture. In both cases, the rupture of the fibers was perpendicular to or at an angle to the longitudinal axis of the fibers. The average lengths of destroyed carbon fibers from the tested processes ranged from 15 to 20 µm and 30 to 60 µm for glass fibers.

## 1. Introduction

Composite systems based on carbon and glass fibers with reactive plastic matrices are, thanks to their mechanical parameters, widely used materials in many industrial areas, starting with the aerospace industry and ending with sports needs [1,2,3]. Composites are predominantly used in engineering structures due to their higher mechanical properties, superior durability, and fatigue performance. Many relevant studies provide not only an extensive scientific background but also state the current state of the issue [4,5,6].

Despite the possibilities of making composite parts so-called “tailor-made,” it is often necessary to use finishing operations, primarily machining processes. For example, in the construction of small aircraft, one hundred thousand holes are drilled into the composite components; in the case of larger aircraft, it is a million holes [7].

The advantage of composite systems is given by using carbon and glass fibers located mainly in a polymer matrix, i.e., materials with entirely different properties, which leads to problems in machining technology caused by the connection of such materials [8,9,10,11,12].

During machining, delamination of layers, destruction of fibers, thermal damage during contact with the tool, crushing of the matrix and fibers, and damage at the interphase interface of the composite occur naturally [13].

The machining of fiber-reinforced composite systems creates non-compact assorted chips, which then break down into dust made up of fibers and a polymer matrix. The morphology and concentration of broken fibers and matrix thus lead to potential hazards for human health and instrumentation. Several toxicological studies have pointed out the dangers associated with the technological process of composite machining systems, but there is still not enough relevant information on this topic [14,15,16,17].

Dust particles created after machining composite parts are made of crushed fibers and polymers and vary in size and concentration in the surrounding environment. The amount of composite dust generated during the machining process depends on the machining process type, material removal rate, tool speed, tool material, and fiber orientation in the composite system. For example, increasing the cutting speed and decreasing the feed rate reduces the number of particles in the surrounding environment [18].

Evidently, the issue is very global and simultaneously includes all parameters entering the technological process, and the conclusions are not always unambiguous. Inhaled dust constitutes approximately 91% of the total number of particles present in the atmosphere, and this percentage represents only 8 to 17% of the actual mass present in the atmosphere. However, even these tiny percentages appear to exceed standard exposure limits. The study showed that almost 90% of the particles in the atmosphere reach the lung alveoli, regardless of the machining conditions and tools used. It is, therefore, logically recommended to use suitable protective equipment and suction devices [19].

During machining, fiber and polymer dust comes into contact with the human body in the following ways: inhalation, ingestion, and skin contact. Toxicological studies on rats and rabbits confirmed an adverse effect on their health, but no significant impact on lung function was recorded. Fibers with a diameter of 7 μm and a length ranging from 20 to 60 μm at 20 mg/m^3^ did not produce systematic health problems in these animals, except for variable respiratory rates. In a study using carbon fibers made from pitch (so-called pitch fibers) with a diameter of 1 to 2 μm and a fiber concentration in the air of 47 or 106 mg/m^3^, transient inflammatory conditions were identified that returned to normal after the exposure ended. In other studies, carbon fiber and epoxy polymer dust were found to cause an apparent histopathological reaction in rats after an intratracheal dose [14,15,19,20,21,22,23].

In works from the 1990s, the authors focused on determining the particle size from the point of view of their aerodynamic distribution on the tool face after milling, drilling, and grinding. It was found that the composite dust on the tool face exhibited dimensions from 7 to 11 μm, a relatively large number of particles had dimensions from 0.8 to 2 μm, and a small amount had dimensions less than 0.2 μm. The sizes of the dust particles and the results of animal studies lead to the opinion that the dust released during the processing of composite materials is fibrogenic for humans, whether it is fibers from PAN or fibers from pitch [15,17,20].

Dust particles created during the machining of composite systems and subsequently detected in the air differ in their dimensions and quantity. The so-called aerodynamic diameter of the particles that are usually released during machining was determined (the aerodynamic diameter of a particle is the diameter of a sphere with a density of 1 g/cm^3^ with the same final velocity caused by the force of gravity in still air as the particle has under the usual conditions of temperature, pressure, and relative humidity). It has been found that particles larger than 10 μm can be trapped in the nasal cavity. Particles with an aerodynamic diameter range of 5 to 10 μm are inhalable and settle in the nasopharynx region. Particles 2 to 5 μm in size can enter the trachea and bronchi; particles smaller than 2 μm can enter the alveoli; and particles smaller than 1 μm quickly enter the bloodstream. In many studies that have addressed this issue, fiber and polymer dusts have been found to cause a definitive histopathological response in the lungs of rats following an intratracheal dose.

Fibrous solid carbon structures with a length-to-thickness (diameter) ratio greater than or equal to 3 μm are respirable and may cause chronic discomfort [15,24,25,26,27,28].

Another potential problem arising in machining fiber composite systems is the effect of the fibers on the skin. Carbon and glass fibers cause dermatitis, rashes, and skin and nose irritation. These problems may appear gradually after exposure to dust, usually disappearing after several months if the exposure to such particles is eliminated [18,20].

The Occupational Safety and Health Administration (OSHA) defines respirable dust as particles that are small enough to enter the nose, upper respiratory tract, and lungs. The National Institute for Occupational Safety and Health (NIOSH) considers fibers less than 3.5 μm in diameter respirable. The Mine Health and Safety Administration (MHSA) considers aerodynamic dust smaller than 5 μm respirable. According to the MHSA, 25% and 90% of dust are respirable if their aerodynamic diameters are equal to or less than 5 μm and 2 μm, respectively. The average concentration should be kept below 15 mg/m^3^. Other publications give limits of 10 mg/m^3^ for total airborne particulate mass and 5 mg/m^3^ for 8 h of exposure [20,29,30,31].

The formation of dust particles in composite systems is influenced by the chosen process, its conditions, and the orientation of the fibers embedded in the polymer matrix. The highest mass concentration of dust occurs when the fibers are distributed at 0° and the lowest at 90° in the tool’s movement direction. At least 50% of the generated particles show an aerodynamic diameter smaller than 0.12, 0.31, 0.21, and 0.35 for milling in the order 0°, 45°, 90°, and 135°. According to the studies, individual processes differ in the production of dust; however, the conditions of implementation of these technological operations and the choice of cutting tools can significantly influence this fact [32,33].

Much of the work published so far is devoted to studying composite systems and dust formation in a simplified view—the movement of the tool at different angles and speeds within a single layer. In practice, composite boards are mainly machined and made up of many layers, and modeling processes are complicated in these cases. Another element, which is considered only marginally, is the fact that the number of dust particles created during machining that are released into the air is determined and monitored. However, much of the destroyed material remains on the work surface [34].

The study aimed to evaluate and compare the dimensions and character of broken carbon and glass fibers and epoxy matrix, which generally remain on the worktop after milling, drilling, and grinding, carried out under selected conditions compared to published work so far, in which the authors dealt with the issue of destroyed fibers that moved in the air and were thus directly health-threatening elements. The obtained results are the basis for further research in machining composite materials and the simultaneous improvement of work safety in these operations. This experimental study followed up on previous work, which was primarily concerned with evaluating the delamination of composite systems and the wear of the tools used during chip machining [35,36,37,38]. The achieved results are essential for industrial practice, health, and safety protection in the machining of composites. Based on the results of this study, a system is developed and constructed for the extraction of dust particles during machining without process media. The chopped fibers of both types can be used as recycled filler in polymer systems after removing the polymer matrix.

## 2. Materials and Methods

Samples of composite materials were prepared from epoxy resin ChS-Epoxy 520 cured with hardener T 0492 in a mass ratio of 100:26 (Districhem, a.s., Ústí nad Labem, Czech Republic) and carbon fabric KC 160 g/m^2^ 3K and glass fabric AEROGLASS 280 g/m^2^ in both cases with a twill weave (Havel Composites CZ, s.r.o., Svésedlice, Czech Republic). Compact samples with dimensions of 200 × 300 × 25 mm were prepared by layering resin-impregnated fabric. A total of 120 and 125 layers of carbon and glass fabric impregnated with epoxy resin in a ratio of 4:6 were used. The material was chosen intentionally for two reasons: in the production of composite parts, textiles are used the most often, and when machining a composite sample with fabric, the destruction of fibers was included both in the longitudinal and transverse directions at an angle. The curing of the pieces took place at a temperature of 22 °C ± 2 °C within 24 h; the airing of the samples was ensured by the vacuum bagging method. The samples were further cured at 50 °C ± 2 °C for 10 h. The produced boards were cut into pieces with dimensions of 90 × 90 × 25 mm, which were used for individual experiments. The values of the elastic modulus of commonly used PAN carbon fibers are 250 to 380 GPa in the direction of the axial axis and 10 to 20 GPa in the direction of the radial axis. Glass fibers have a modulus of elasticity of about 80 GPa. The modulus of elasticity of epoxy resins is 3 to 6 GPa. The mechanical parameters of the prepared samples were not evaluated in this study.

Chip machining, milling, drilling, and grinding were selected for fiber destruction in epoxy resin/carbon fiber and epoxy resin/glass fiber composite systems. Milling was performed on an FNG 32 milling machine (TOS Olomouc, s.r.o., Olomouc, Czech Republic) using a cylindrical end mill with a diameter of 80 mm and seven replaceable inserts, TNGX 100404SR-F (Dormer Pramet, s.r.o., Šumperk, Czech Republic). For milling, the rotation speed *n* = 1000 rpm, depth of engagement a_p_ = 1 mm and 2 mm, and feed rate v_f_ = 100 mm/min were selected. Drilling was carried out on a FNG 32 machine (TOS Olomouc, s.r.o., Olomouc, Czech Republic); HSS-Co DIN 338 drills with a diameter of D = 5 mm and 9.8 mm with a tip angle of 118 ° were used for the experiment (Oren, s.r.o., Údlice, Czech Republic). The following cutting conditions were chosen for the experiment: revolutions *n* = 500, 1000, 1500, and 2000 rpm, and shift per revolution f_n_ = 0.2 mm/rev. Grinding was carried out on a TOS BPH 320 A/100 surface grinder (TOS VARNSDORF, a.s., Varnsdorf, Czech Republic), with a grinding wheel T1 250 × 32 × 76 98A60K9V40 421188 (TYROLIT CEE, a.s., Benátky nad Jizerou, Czech Republic). The rotation speed *n* = 2400 rpm, the feed rate v_f_ = 200 mm/min, and the engagement depth a_p_ = 0.2 mm were selected. The cutting conditions were determined according to industry practice. No process medium was used in the experiments.

A standard laboratory furnace fires epoxy resin from chip samples from individual machining processes. The burning temperature was 500 °C ± 10 °C, and the burning time was 2 h.

A Nikon EPIPHOT 200 optical microscope (Nikon, Minato, Japan) with the NIS-elements 5.02 program was used to evaluate the micrometer lengths of the fibers. The electron microscope TESCAN MIRA 3 (TESCAN Holding, Brno, Czech Republic) was used to monitor sub-micrometer fiber lengths, assess the nature of fiber fractures, and describe the nanometer and sub-micrometer particles produced during the machining process. The surface of the samples was sputtered with a layer of Pt-Pd (Quorum Q 150R ES) with a thickness of 2 to 4 nm.

## 3. Results

**Figure 1:**
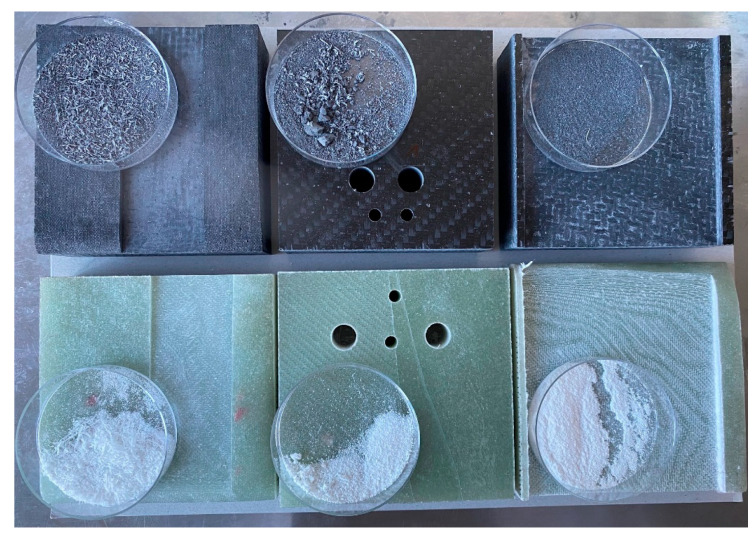
Image of prepared composite samples and chips from them during the machining processes of milling, drilling, and grinding (from left to right). Carbon fiber composite samples (upper row), glass fiber composite samples (lower row).

Only the chips of carbon/glass fiber composites created by the tool, which remain on the work surface next to the machined body and are subsequently thrown into the waste, were selected for evaluation (Figure 1). Particles that are potentially released into the air were not monitored in this study. The following evaluation criteria were chosen: the length of the fibers, or the size and shape of the particles formed during the destruction of the fibers, the nature of the fiber fracture, the angle at which the fibers break, the formation of cracks in the fibers, and the splitting of the fibers in the direction of the fiber axis. The following results document the experimental procedure’s progress in determining the parameters depending on the chosen type of chip machining and its conditions.

**Figure 2:**
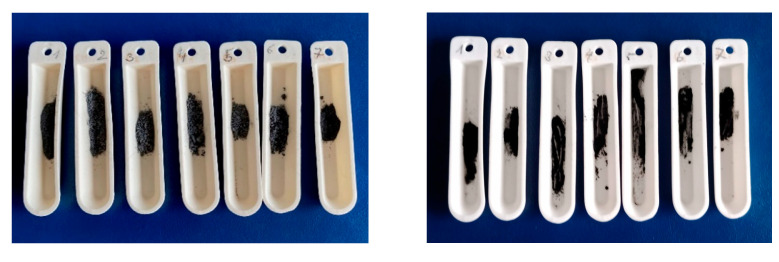

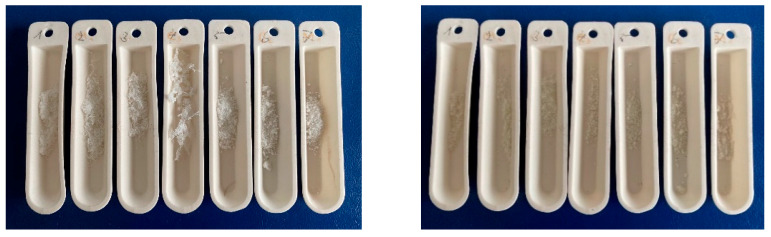
Illustrative image of chips generated during the selected machining process (**left**) and after removing the epoxy matrix by firing it (**right**) for carbon fiber (**top**) and glass fiber (**bottom**) systems.

Electron microscope analyses were performed, which provided information on the nature of the fractures of both types of fibers, the size of the angles at which fiber fracture/destruction occurs, the nature of cracks in the fibers, and the adhesion between the fibers used and the epoxy matrix. During all the selected machining processes, the destruction of the reinforcing fibers and the crushing of the epoxy matrix into particles comparable to the particles formed from the fibers occurred, making the analysis process difficult. Chips from all the samples taken were burned in air at a temperature of 500 °C ± 10 °C for 2 h and then weighed (Figure 2). From the analyzes carried out, it was found that the actual amount of fibers in the polymer matrix was in the ratio 3.8:5.2. 

After burning the samples, analyzes were carried out on an optical and then repeatedly on an electron microscope, the aim of which was to analyze the lengths of the destroyed glass and carbon fibers, the nature of the fractures, and at the same time to find out basic information about the relationship between the destruction of the fibers and the used machining technology. Based on the measurements made, the dependencies of the frequency of occurrence on the lengths of the destroyed fibers concerning the technology used were compiled.

### 3.1. Milling of Carbon Fiber Composite Systems

**Figure 3:**
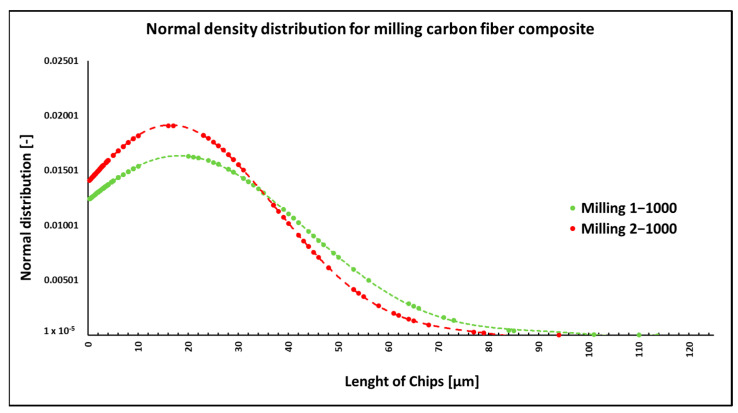
Dependencies of frequency of occurrence and lengths of destroyed fibers concerning milling technology.

**Figure 4:**
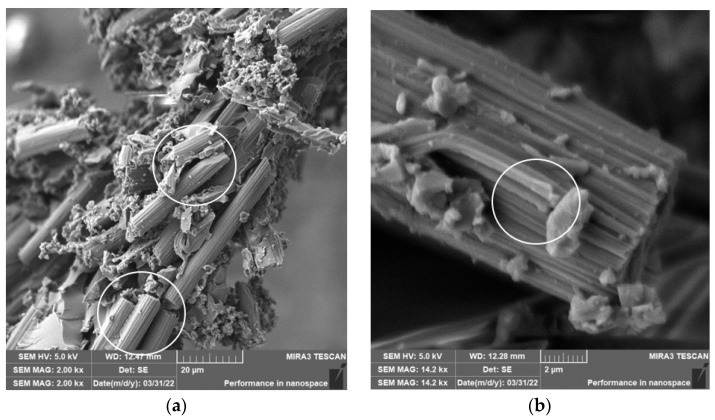
Carbon fibers in an epoxy matrix. (**a**) Detailed image of a chip after milling (*n* = 1000 rpm, a_p_ = 1 mm, and v_f_ = 100 mm/min) with broken carbon fibers and matrix particles—in white circles are directions of fractures—perpendicular and at an angle to the fiber axis. (**b**) Close-up of the surface longitudinal failure of carbon fiber with matrix particles in the white circle.

**Figure 5:**
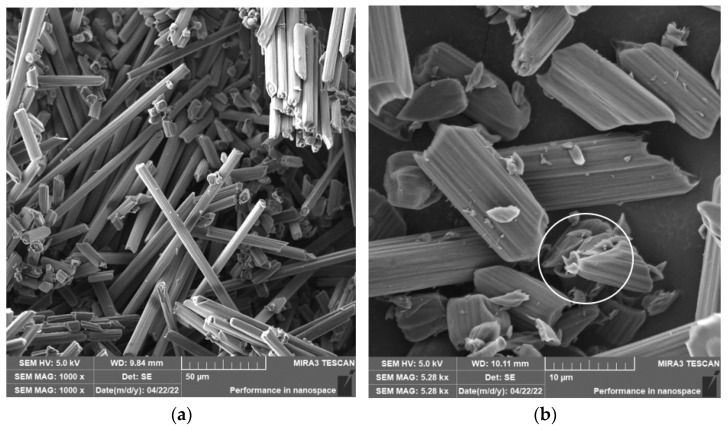
Carbon fibers after removing the epoxy matrix. (**a**) Image of carbon fibers formed after milling (*n* = 1000 rpm, a_p_ = 2 mm, and v_f_ = 100 mm/min) with an example of length variability. (**b**) A close-up of the nature of the direction of fractures and the sizes of the carbon fiber fragments after milling (*n* = 1000 rpm, a_p_ = 1 mm, and v _f_ = 100 mm/min) in a white circle.

During the milling of composite samples under the given cutting conditions (*n* = 1000 rpm, a_p_ = 1 mm, and v_f_ = 100 mm/min), carbon fibers were shortened to lengths ranging from 0.3 µm to 115 µm, undercutting conditions (*n* = 1000 rpm, a_p_ = 2 mm, and v_f_ = 100 mm/min) produced fibers with a length ranging from 0.3 µm to 94 µm (Figure 3). In both conditions, fiber splitting occurred both perpendicular to the fiber axis and at an angle of 30° to 60° to the fiber axis (Figure 4). The particle size of the polymer matrix generated during milling ranged from units of micrometers to hundreds of micrometers. Carbon fibers were broken by a tough fracture in all machining cases (Figure 4a) are typical cases of such fractures. After firing the polymer matrix, small particles formed by destroying carbon fibers with dimensions in units of micrometers were visible. Even smaller particles, at the level of hundreds of nanometers, were also identified (Figure 5). From the picture in Figure 4b, atypical destruction of the carbon fiber is also visible—the longitudinal part of the fiber—“bundle of graphene layers”—parallel to the axis of the fiber has been torn off.

NIS-ELEMENTS 5.02 analysis was used to determine fiber fragment lengths after the thermal removal of crushed epoxy resin particles. It was found that the shortest fibers that are generated using this technology have a size of 0.3 µm, the longest fibers have a length of 114 µm, and the average length is 19 µm (Figure 3). The conditions chosen for this technology (number of revolutions per minute and engagement depth) do not have a fundamental effect on the length of the generated fragments.

### 3.2. Drilling of Carbon Fiber Composite Systems

**Figure 6:**
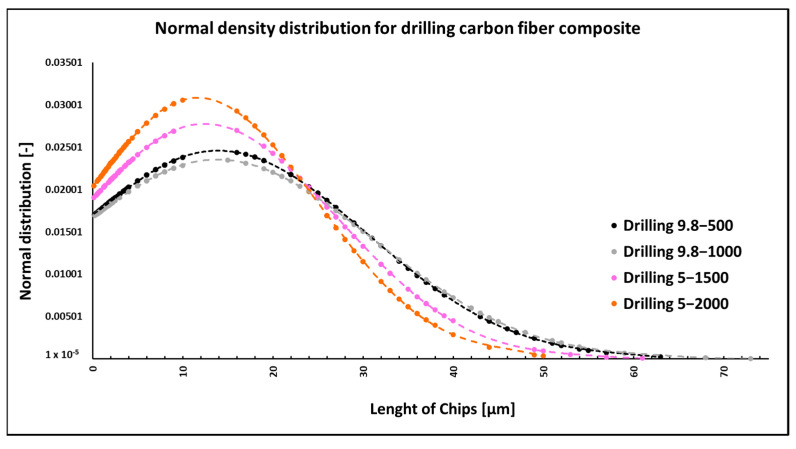
Dependencies of frequency of occurrence and lengths of destroyed fibers concerning drilling technology.

**Figure 7:**
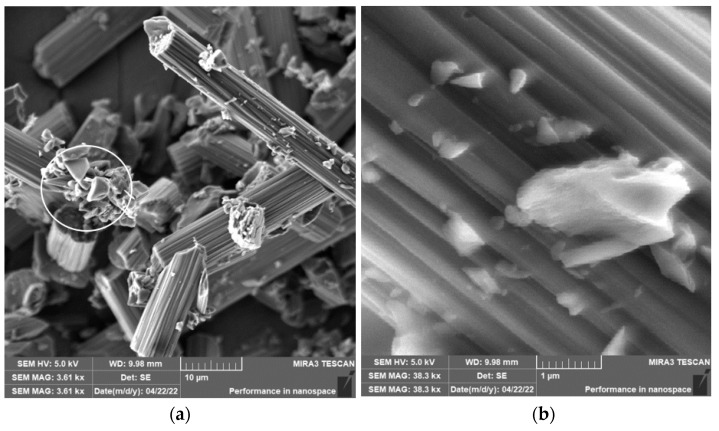
Carbon fibers after removing the epoxy matrix. (**a**) Image of carbon fibers and their fragments formed after drilling in a white circle (*n* = 1500 rpm, D = 5.0 mm, and f_n_ = 0.2 mm/min). (**b**) A close-up of the surface of a carbon fiber with sub-micrometer particles formed during its destruction.

**Figure 8:**
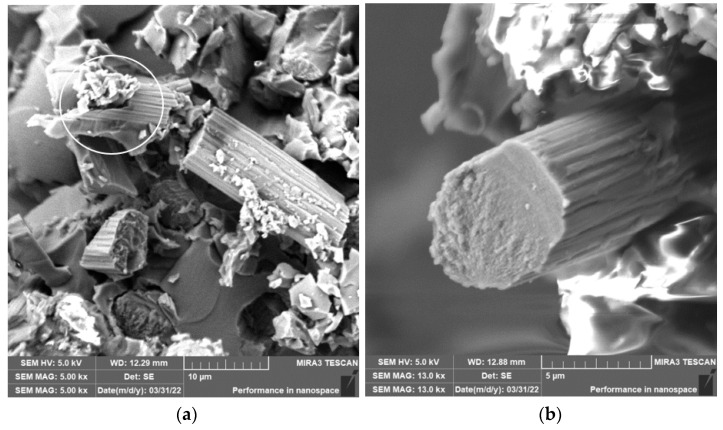
Carbon fibers in an epoxy matrix. (**a**) Image of carbon fiber fragments after drilling (*n* = 1000 rpm, D = 9.8 mm, and f_n_ = 0.2 mm/min) with compact matrix and matrix particles visible in a white circle. (**b**) A detailed image of the fracture surface character of the carbon fiber after drilling (*n* = 1500 rpm, D = 9.8 mm, and f_n_ = 0.2 mm/min).

When drilling composite samples under cutting conditions (*n* = 1000 rpm, D = 9.8 mm, and f_n_ = 0.2 mm/min), carbon fibers were shortened to lengths ranging from 0.25 µm to 75 µm, undercutting conditions (*n* = 1500 rpm, D = 5.0 mm, and f_n_ = 0.2 mm/min) produced fibers with a length ranging from 0.12 µm to 60 µm (Figure 6). In both conditions, fiber shortening occurred more often perpendicular to the fiber axis than at an angle compared to milling (Figure 4); the ends of the fibers also broke off (Figure 7a). After burning the polymer matrix, particles formed by the destruction of carbon fibers with dimensions on the level of hundreds of nanometers (Figure 7b) and at the same time small particles from units to tens of micrometers (Figure 8a) were easily identifiable. The size of the polymer matrix particles generated during milling varied from units of micrometers to tens of micrometers. The carbon fibers were broken by a tough fracture, as seen in the pictures (Figure 7 and Figure 8b). 

NIS-ELEMENTS 5.02 analysis was used to determine fiber fragment lengths after the thermal destruction of epoxy resin particles. It was found that the shortest fibers that are generated using this technology have a size of 0.12 µm, the longest fibers have a length of 75 µm, and the average length is 13 µm (Figure 6). The conditions of the selected technology (drill diameter and revolutions) have an effect on the size of the generated fragments.

### 3.3. Grinding of Carbon Fiber Composite Systems

**Figure 9:**
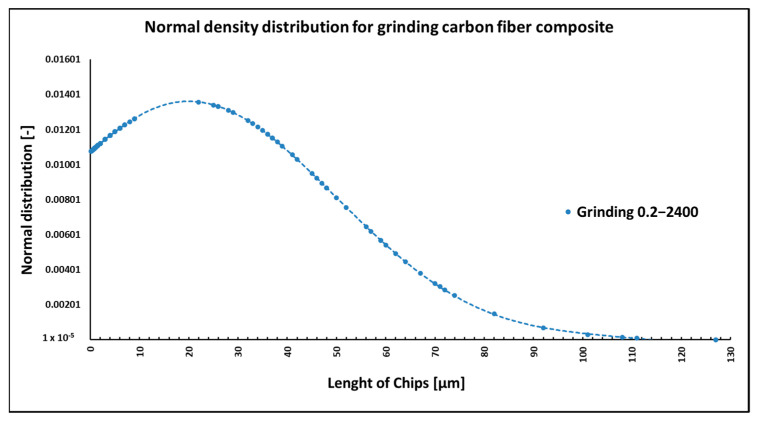
Dependencies of frequency of occurrence and lengths of destroyed fibers concerning grinding technology.

**Figure 10:**
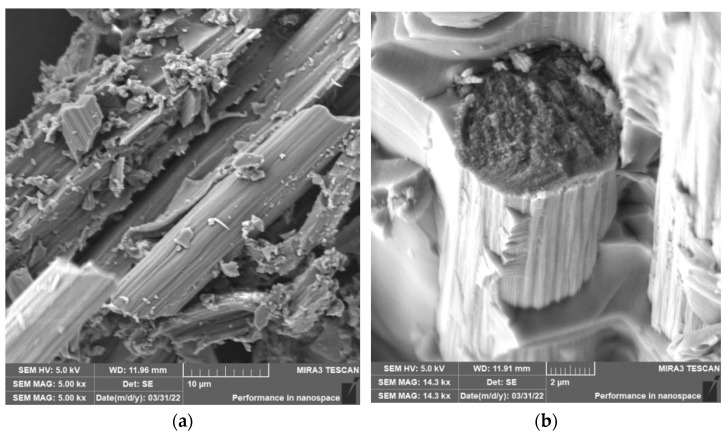
Carbon fibers in an epoxy matrix. (**a**) Image of carbon fibers and their fragments after grinding (*n* = 2400 rpm, a_p_ = 0.2 mm, and v_f_ = 200 mm/min). (**b**) Close-up of the fracture surface of carbon fiber surrounded by an epoxy matrix.

**Figure 11:**
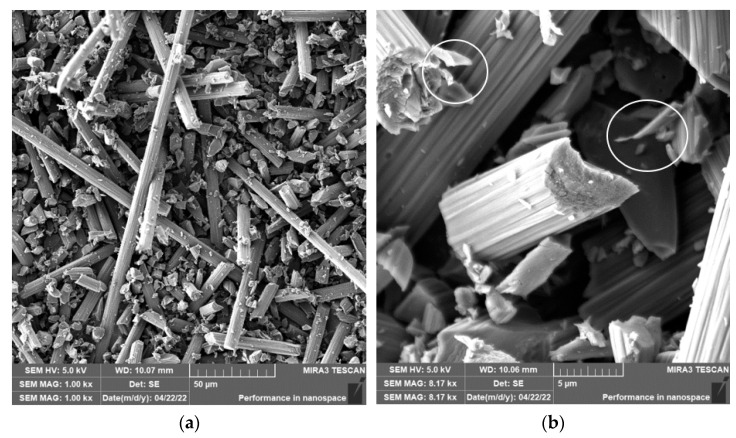
Carbon fibers after removing the epoxy matrix. (**a**) Image of carbon fibers and their fragments formed after grinding (*n* = 2400 rpm, a_p_ = 0.2 mm, and v_f_ = 200 mm/min). (**b**) A detailed image of carbon fibers and their fragments in a white circle, including the nature of fracture surfaces.

When grinding composite samples under cutting conditions (*n* = 2400 rpm, a_p_ = 0.2 mm, and v_f_ = 200 mm/min), the fibers were shortened to lengths ranging from 0.15 µm to 127 µm (Figure 9). Fibers have shortened perpendicular to the fiber axis during grinding (Figure 10 and Figure 11a), at an angle similar to milling (Figure 4 and Figure 5). The size of the polymer matrix particles released during grinding ranged from units of micrometers to tens of micrometers; despite the fact that the sample was destroyed by machining, very good adhesion of the matrix to the carbon fibers was evident. As in previous cases, the carbon fibers broke through a tough fracture (Figure 10 and Figure 11b). After burning the polymer matrix, tiny particles of carbon fiber resulting from their destruction with dimensions ranging from hundreds of nanometers to tens of micrometers were easily identifiable (Figure 10 and Figure 11). In the picture in Figure 11b, very thin layers released from the carbon fibers are visible.

NIS-ELEMENTS 5.02 analysis was used to determine fiber fragment lengths after chip burning, which removed the epoxy polymer. It was found that the shortest fibers generated using this technology have a length of 0.15 µm, the longest fibers have a size of 130 µm, and the average length is 20 µm (Figure 9). Other process conditions were not experimentally evaluated within the grinding technology.

### 3.4. Milling of Fiberglass Composite Systems

**Figure 12:**
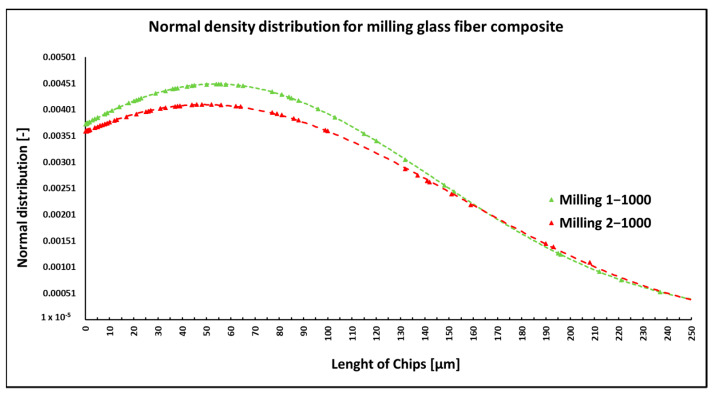
Dependencies of frequency of occurrence and lengths of destroyed fibers concerning milling technology.

**Figure 13:**
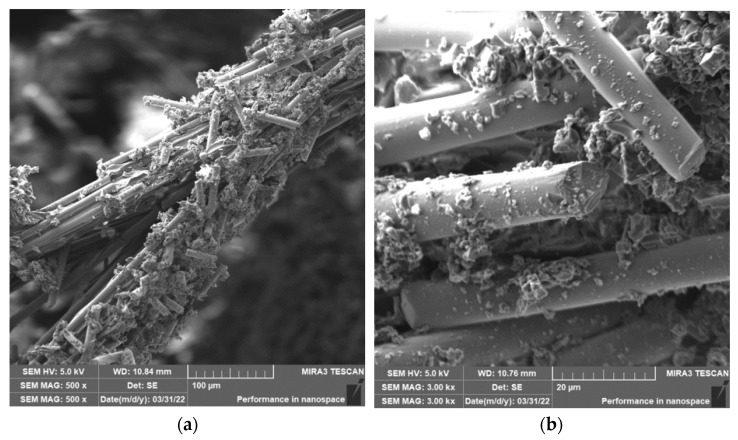
Glass fibers in epoxy resin. (**a**) Image of the chip after milling (*n* = 1000 rpm, a_p_ = 1 mm, and v_f_ = 100 mm/min) with broken glass fibers and matrix particles. (**b**) Close-up of broken glass fibers with matrix particles.

**Figure 14:**
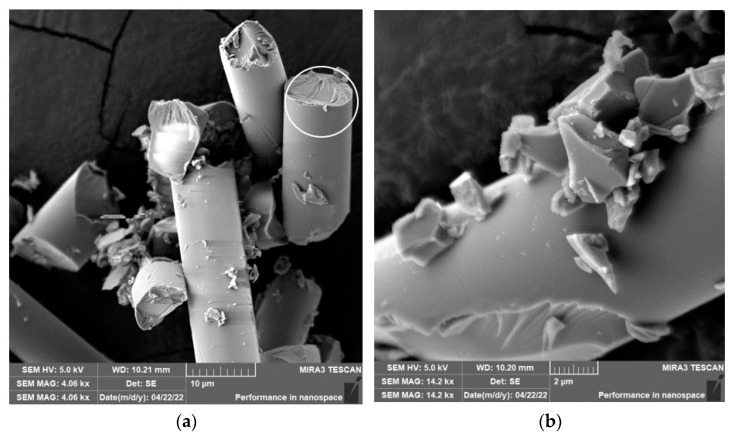
Glass fibers after removing the epoxy matrix. (**a**) Image of glass fibers and fragments created after milling (*n* = 1000 rpm, a_p_ = 2 mm, and v_f_ = 100 mm/min) with a characteristic brittle fracture in a white circle. (**b**) Close-up of glass fibers and their fragments.

During milling of composite samples at cutting conditions (*n* = 1000 rpm, a_p_ = 1 mm, and v_f_ = 100 mm/min), fibers were shortened to lengths ranging from 0.07 µm to 622 µm. At cutting conditions (*n* = 1000 rpm/min, a_p_ = 2 mm, and v_f_ = 100 mm/min), fibers with a length ranging from 0.14 µm to 683 µm were produced (Figure 12). In both conditions, fiber breakage mainly occurred perpendicular to the fiber axis but less so at an angle to the fiber axis (Figure 13). The size of the polymer matrix particles generated during milling ranged from units of micrometers to tens to hundreds of micrometers, similar to the previous case when machining carbon fiber composite systems. Glass fibers broke by a brittle fracture in all cases of machining. After the firing of the polymer matrix, small particles resulting from the destruction of glass fibers with dimensions ranging from units to tens or hundreds of micrometers were primarily visible (Figure 14a). Smaller particles, at tens to hundreds of nanometers, were also identified (Figure 14b).

NIS-ELEMENTS 5.02 analysis was used to determine fiber fragment lengths after the thermal removal of the epoxy polymer. It was found that the shortest fibers generated using this technology have a length of 0.07 µm, the longest fibers are 683 µm, and the average length is 52 µm (Figure 12). The conditions of the selected technology (speed and depth of engagement) do not fundamentally affect the length of the generated fragments.

### 3.5. Drilling of Fiberglass Composite Systems

**Figure 15:**
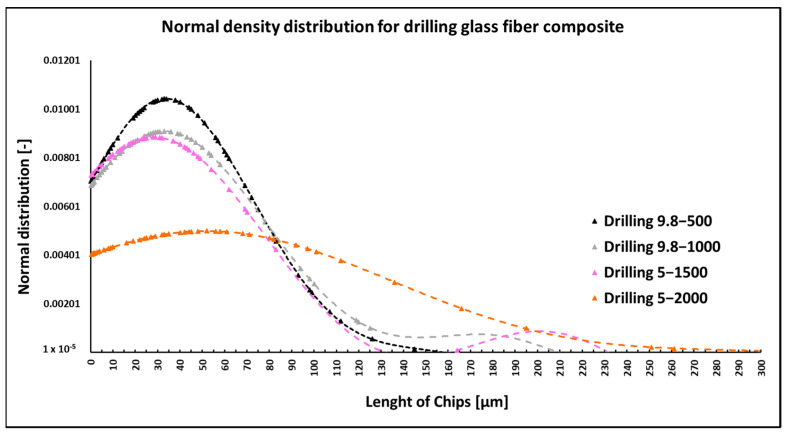
Dependencies of frequency of occurrence and lengths of destroyed fibers concerning drilling technology.

**Figure 16:**
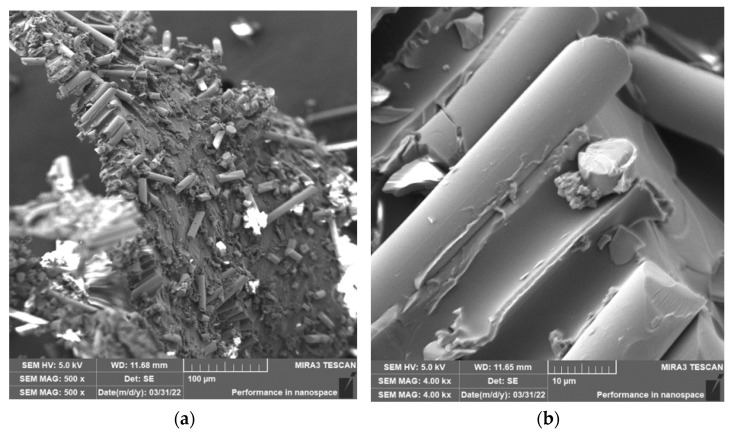
Glass fibers in epoxy resin. (**a**) Detailed chip image after drilling with broken glass fibers and matrix particles (*n* = 1500 rpm, D = 5.0 mm, and f_n_ = 0.2 mm/min). (**b**) Close-up of glass fibers with typical fractures.

**Figure 17:**
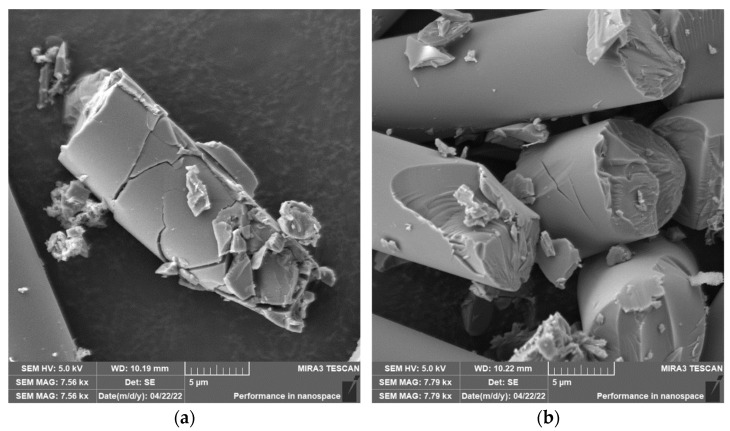
Glass fibers after removing the epoxy matrix. (**a**) Image of glass fiber destruction after drilling (*n* = 1000 rpm, D = 9.8 mm, and f_n_ = 0.2 mm/min). (**b**) A close-up of the characteristic fracture surfaces of glass fibers after drilling (*n* = 1500 rpm, D = 5.0 mm, and f_n_ = 0.2 mm/min).

When drilling composite samples under cutting conditions (*n* = 1500 rpm, D = 5.0 mm, and f_n_ = 0.2 mm/min), glass fibers were shortened to lengths ranging from 0.3 µm to 267 µm, undercutting conditions (*n* = 1000 rpm, D = 9.8 mm, and f_n_ = 0.2 mm/min) produced fibers with a length ranging from 0.26 µm to 227 µm (Figure 15). Under both conditions, fiber splitting occurred mainly perpendicular to the fiber axis (Figure 16 and Figure 17). The size of the polymer matrix particles generated during drilling ranged from units of micrometers to tens of micrometers. A brittle fracture broke the glass fibers; an example is in Figure 17. After firing the polymer matrix, small particles formed by destroying glass fibers with sizes ranging from units to tens of micrometers were visible. Sub-micrometric particles were also identified (Figure 17).

NIS-ELEMENTS 5.02 analysis was used to determine fiber fragment lengths after the thermal removal of the epoxy polymer. It was found that the shortest fibers generated using this technology have a length of 0.25 µm, the longest 398 µm, and an average length of 37 µm (Figure 15). In three cases, there is no significant influence of the drilling conditions (drill diameter, revolutions, and displacement per revolution) on the length of the generated fiber fragments. A change was noted when drilling with a higher speed and a smaller drill diameter. The distribution of the frequency of occurrence depending on the length of the destroyed fibers does not show a significant maximum as in previous measurements under other technological conditions.

### 3.6. Grinding of Glass Fiber Composite Systems

**Figure 18:**
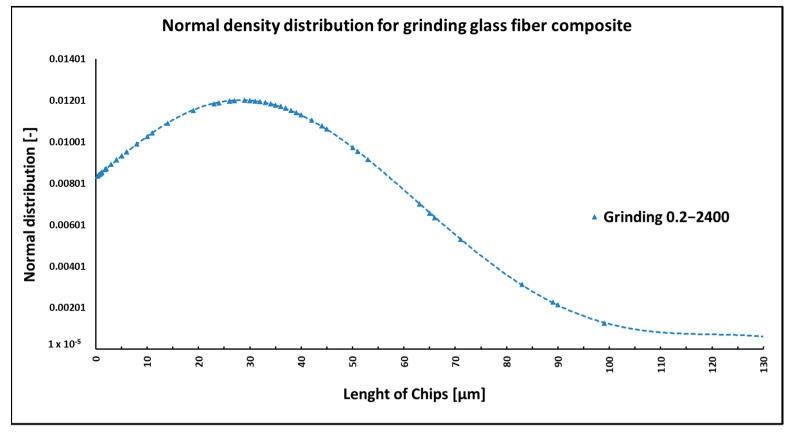
Dependency of frequency of occurrence and lengths of destroyed fibers concerning grinding technology.

**Figure 19:**
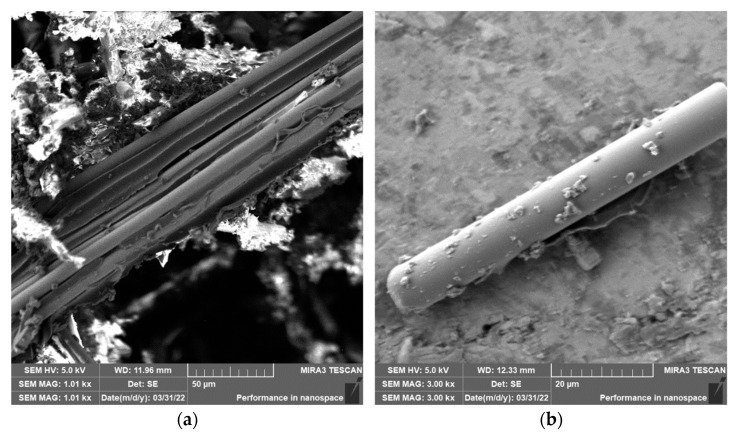
Glass fibers in epoxy resin. (**a**) Image of a bundle of glass fibers and matrix particles after grinding (*n* = 2400 rpm, a_p_ = 0.2 mm, and v_f_ = 200 mm/min). (**b**) Close-up of the glass fiber and matrix particles.

**Figure 20:**
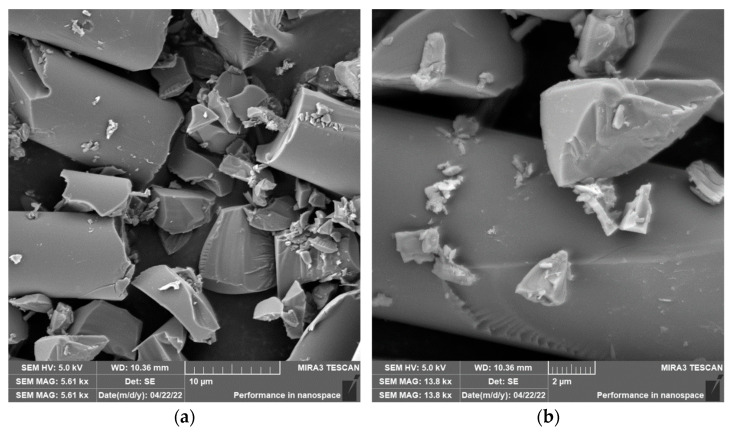
Glass fibers after removing the epoxy matrix. (**a**) Image of destroyed glass fibers after grinding (*n* = 2400 rpm, a_p_ = 0.2 mm, and v_f_ = 200 mm/min). (**b**) Close-up of sub-micrometer glass fiber particles.

Grinding was carried out with cutting conditions (*n* = 2400 rpm, a_p_ = 0.2 mm, and v_f_ = 200 mm/min). During the grinding of the glass fiber composite samples produced particles ranging in size from 0.31 µm to 220 µm (Figure 18). In Figure 19a, it can be seen that during the grinding of the fiberglass composite, chips were formed in the form of long bundles of matrix-coated fibers. Figure 19b shows a single fiber with pieces of matrix adhered to it. After burning the polymer matrix, tiny particles of glass fiber resulting from their destruction with dimensions ranging from hundreds of nanometers to tens of micrometers were easily identifiable (Figure 20a,b).

NIS-ELEMENTS 5.02 analysis was used to determine fiber fragment lengths after the thermal removal of the epoxy matrix. It was found that the shortest fibers generated using this technology have a size of 0.310 µm, the longest fibers have a length of 220 µm, and the average length is 29 µm (Figure 18). Multiple technological conditions were not evaluated in this experiment.

The quantitative relationship between parameters such as the length of the destroyed fibers, the nature of the fracture, and the size of the fractions of individual dimensions can be summarized as follows: carbon fibers are crushed to smaller dimensions than glass fibers under the used machining technologies and conditions. The observed sizes of the destroyed carbon fibers range from hundreds of nanometers to about 130 μm; in the case of milling, they have the most significant representation of fibers with an average length of 19 µm, in drilling 13 µm, and in grinding 20 µm. The fracture is always at an angle to the fiber axis or perpendicular to the axis. The technology and machining conditions do not significantly affect the length of the resulting particles or the nature of the fracture. Destroyed glass fiber particles range from tens/hundreds of nanometers to hundreds of micrometers. In the case of milling, fibers with an average length of 52 µm have the most significant representation in the cases of drilling with 37 µm and grinding with 29 µm. The fibers break brittlely, at an angle, or perpendicularly. The lengths of broken carbon fibers are almost half those of glass fibers; the angle of refraction is analogous. The explanation for the significantly lower values of the sizes of the destroyed carbon fibers lies in their morphology, i.e., in the different values of Young’s modulus of elasticity in the axial direction, where it reaches between 250 and 380 GPa, and in the radial direction, in which it has values of 10–20 GPa. In contrast, the modulus of elasticity of glass fibers, as an isotropic amorphous material, reaches tens of GPa in all directions. When machining layered composite systems, the tool will interact with the fiber in the axial and radial directions, so it is clear that in the radial direction, the carbon fiber is more fragile than the glass fiber, and therefore its destruction will be easier. As part of the implemented chip machining experiment on multilayer samples made of carbon and glass fabrics, the technology and machining conditions did not significantly affect the average lengths of the destroyed fibers. For a better understanding of the processes and effects, it would probably be more appropriate to work only with a single-layer material of parallel fibers, where the effects of the machining conditions would be noticeable. In the case of layered materials, there is a combination of effects, stress, and deformation of the fibers at an angle, and the effects of conditions and technologies become challenging to grasp. When drilling a multilayer sample with carbon fibers, the lowest average values of the lengths of the destroyed fibers were measured, probably also because the intruding chip could continue to grind in the drilled hole.

## 4. Discussion

When machining composite materials with carbon and glass fibers in epoxy resin, chips of typical shapes and sizes were formed according to the chosen technology (milling, drilling, and grinding) (Figure 1). Chips of a more or less compact nature were composed of epoxy resin and reinforcing fibers and fell into smaller fragments after handling. The fragments were formed by an epoxy matrix crushed into larger or smaller particles with dimensions of units up to hundreds of micrometers, which had greater or lesser adhesion to the reinforcing fibers. Both glass and carbon fibers were chopped to lengths ranging from hundreds of nanometers to tens of micrometers. The nature of fiber failure generally varied according to the type of fibers used—carbon fibers showed a tough fracture, and glass fibers showed a brittle fracture. The fiber length and the number of individual fractions varied according to the machining method and conditions.

The length of the carbon fibers, which were separated from the chips and subsequently analyzed after the milling process, ranged from 0.3 µm to 115 µm Figure 3. Carbon fibers were broken both at an angle of 30° to 60° towards the fiber axis and perpendicular to the fiber axis (Figure 4 and Figure 5); in isolated cases, the fiber was damaged in the direction of its longitudinal axis (Figure 4b). There was also the formation of tiny parts of the fibers—fragments, which were observed as adhered particles on the surface of the carbon fibers (Figure 5).

In the case of milling composite samples with glass fibers, the length of the fibers ranged from 0.065 µm to 683 µm (Figure 12). The fibers broke perpendicularly and at an angle to the fiber’s longitudinal axis. The images from the electron microscope showed many small particles created by the destruction of fragile glass fibers, with dimensions in units of micrometers.

The length of the carbon fibers produced during the drilling process ranged from 0.12 µm to 73 µm (Figure 6); the fibers were broken similarly as in the case of milling, i.e., at an angle of 30° to 60° towards the axis of the fiber, so perpendicular to the axis (Figure 7 and Figure 8). After removing the particles of the epoxy matrix by burning, tiny particles with sizes below 1 µm, i.e., hundreds of nanometers, which adhered to the surface of the destroyed carbon fibers, were visible on the microscopic images. The smallest carbon fiber particles ever identified were in the drilling process. This size of the particles is already very problematic because, according to the literature, these are inhalable particles that can enter both the alveoli and the bloodstream [18,19].

In the case of composite samples with glass fibers, particles with dimensions from 0.25 µm to 398 µm were formed (Figure 15). Glass fibers were broken by a brittle fracture perpendicular to the axis of the fiber (Figure 17). Sub-micrometric particles have also been identified.

When grinding composite samples with carbon fibers, relatively long fibers with more than 120 µm were released, compared to the assumption. The length of the carbon fibers produced during grinding ranged from 0.14 µm to 127 µm (Figure 9). As in the previous cases, the fracture of the fibers was tough and at an angle perpendicular to the direction of the axis of the fiber. Even in the case of grinding, particles with dimensions below 1 µm were identified, as were more or less flat formations that had to be chipped off the carbon fiber surface (Figure 11). Formations of a similar nature were also identified in a previous experimental study [38].

In the case of composite samples with glass fibers, particles with dimensions ranging from 0.31 µm to 220 µm were formed (Figure 18). Glass fibers were broken by a brittle fracture perpendicular to the axis of the fiber (Figure 19 and Figure 20). As can be seen from the picture (Figure 20), particles created by destroying glass fibers have random, sharp-edged shapes. Sub-micrometric particles adhering to the surface of the fibers were also identified.

Parameters such as the length of the destroyed fibers, the nature of fiber breakage, and the proportion of fractions of individual sizes are influenced by the behavior between the composite sample and the tool, material composition and structure, process conditions—the type of machining technology used, the angle of the tool face, and cutting conditions. In composite systems, reinforcing fibers are the dominant component of the material system due to their characteristic properties. At the same time, an important parameter is the way the fibers are placed in relation to the direction of movement of the cutter. Here, the polymer serves as a binder and mediates the transfer of forces between the fibers. Violation of the system, especially fiber breakage, must therefore be analyzed from the point of view of stress on the fibers, which leads to their subsequent destruction [34].

When machining in the direction parallel to the direction of the fibers (0°), the tool moves at a certain speed, and the interaction between the tool face and the material generates the main cutting force. The cutting force is decomposed into a force parallel to the fiber direction and a force perpendicular to the fiber direction. A force perpendicular to the fiber creates compressive stress on the fiber; the fibers are pushed, bend to both sides (buckling), cracks appear in the tensile stress points in the fibers, and the fibers break. The fragile polymer matrix surrounding the fibers is also stressed, cracking and breaking into small particles.

When machining perpendicular to the fiber’s direction (90°), the main cutting force is perpendicular to the fiber axis. It can be decomposed into the cutting force in the fiber plane and the cutting force perpendicular to the fiber plane. The fibers are stressed by pressure at the point of contact with the tool and can form cracks; on the opposite side of the fiber, there is tension and subsequent breakage. The polymer matrix is subjected to pressure in front of the cutter, and cracks are formed. The fragile matrix is crushed into small particles.

When machined at an angle (45°/135°), the cutting force is distributed into a cutting force perpendicular to the fiber direction and a cutting force parallel to the fiber direction. Fibers are affected by compressive and tensile forces—fibers in direct contact with the tool are stressed by pressure while stretched on the other side. Cracks appear, and fibers are pushed, pulled, and bent simultaneously. Cracks formed on one side of tensile stress propagate easily, while cracks on the other side of compressive stress propagate poorly. As a result of these forces, the fibers crack and split. At the same time, the fragile resin is destroyed under pressure, tension, and bending; the matrix carries compressive stress and creates compressive deformation. As a result, cracks propagate perpendicular to the direction of the fibers in the interface region, and in some cases, longitudinal destruction of the fibers may also occur [19].

As the thread-tool relationship is modeled, these considerations are a very simplified version of the actual situation. Given that, in the case of composite systems created as standard from fabric layers or from strands of fibers containing thousands of individual filaments, which are interconnected by various types of textile weaves (in the case of the experiments presented here, twill weave) and stored in a polymer matrix of entirely different physical, chemical, and mechanical parameters compared to fibers, there is a voltage influence not only in one direction but also in area and volume, mainly if a high-quality interphase interface is ensured.

## 5. Conclusions

This work aimed to analyze the nature of chips that remain on the work surface after composite machining systems based on carbon or glass fibers in an epoxy matrix. This material is often regarded as ordinary waste, and its disposal is not given due attention, despite handling particles that are considered respirable and potentially very dangerous due to their size [18,19]. From the experiments carried out as part of this study, it was found that:

- During the selected machining processes implemented on the prepared composite samples, the primarily occurring chips fragmented into smaller particles—dust, which is made up of fibers and a polymer matrix—appears. Both the polymer matrix and the destroyed fibers showed potentially harmful dimensions for health. However, the stability of reinforcing fibers, both carbon and glass, is greater than the stability of the polymer used;
- The dimensions of destroyed carbon fibers range from hundreds of nanometers to about 130 μm. The minor average fiber lengths are generated during drilling (13 µm), the longest; on the contrary, is generated during grinding (20 µm), fiber fracture is tough perpendicular to the fiber axis or at an angle;
- The dimensions of destroyed glass fibers range from tens/hundreds of nanometers to hundreds of micrometers. The minor average fiber lengths are generated during grinding (29 µm), and the longest, on the contrary, during milling (52 µm). Fiber breakage is fragile, perpendicular to the fiber axis or at an angle;
- In both cases, machining conditions and technologies had no significant influence. Their mechanical parameters considerably impact the length of the destroyed fibers.
- In both cases, tiny glass and carbon particles with dimensions below 2 µm and hundreds of nanometers are formed. From a health point of view, particles smaller than 2 µm are potentially the most harmful. Particles of these dimensions tend to penetrate the respiratory tract; the smallest particles can reach the alveoli or bloodstream [18,19]. In industrial companies, workers processing composite materials use FFP3 respirators, which have the lowest allowed overall efficiency of 98% at a size of 0.36 µm;
- From the presented analysis, it is evident that tiny particles, potentially hazardous to health, were found even in the waste that remains on the work surface after machining. Particles released into the air during the processing of composite materials, even those that stay on the work surface, have dimensions comparable to those of bacteria and viruses and can quickly enter the human body [39,40].

## Data Availability

The authors confirm that the data supporting the findings of this study are available within the article.
